# Diesel Particulate Matter (DPM)-Induced Metabolic Disruption in Mice Is Mitigated by Sodium Copper Chlorophyllin (SCC)

**DOI:** 10.3390/nu17040717

**Published:** 2025-02-18

**Authors:** Jack H. Radford, Ethan P. Evans, Isaac T. Edwards, Juan A. Arroyo, Benjamin T. Bikman, Paul R. Reynolds

**Affiliations:** Department of Cell Biology and Physiology, Brigham Young University, Provo, UT 84602, USA

**Keywords:** chlorophyllin, inflammation, mitochondria, diesel particulate matter

## Abstract

Background/Objectives: The increasing prevalence of metabolic disorders underscores the need for effective interventions to mitigate environmental stressors such as diesel particulate matter (DPM), a major urban air pollutant. DPM is composed of fine carbonaceous particles that can induce systemic inflammation. This phenomenon results in metabolic dysfunction such as adipocyte hypertrophy, insulin resistance, and mitochondrial impairment in body tissues. Methods: This study investigated the impact of DPM exposure on murine lung, skeletal muscle, and adipose tissues and evaluated the protective effects of supplementation with sodium copper chlorophyllin (SCC). Results: Compared to controls, DPM-exposed mice exhibited significantly elevated oxidative stress markers (* *p* ≤ 0.05), systemic pro-inflammatory cytokines including TNF-α, MCP-1, IL-6, and IL-1β (* *p* ≤ 0.05), and adipocyte hypertrophy of both subcutaneous and visceral fat depots, supporting prior findings of DPM-induced metabolic dysfunction. SCC supplementation restored pulmonary ATP levels (* *p* ≤ 0.05), significantly reduced ROS production in lung and muscle tissue (* *p* ≤ 0.05), and significantly attenuated DPM-induced inflammatory cytokine secretion (* *p* ≤ 0.05), while lessening DPM-induced adipocyte hypertrophy. Conclusions: These effects highlight the antioxidant and anti-inflammatory potential of SCC, which likely mitigates systemic metabolic compromise by modulating mitochondrial function and inflammatory pathways. This study further demonstrated that SCC supplementation may be an effective intervention for alleviating the adverse effects of DPM exposure on metabolic and inflammatory compromise. Additional research may clarify a role for SCC in reducing systemic health risks associated with air pollution and offer a foundation for future translational research in human populations exposed to environmental pollutants.

## 1. Introduction

Metabolic complications have increasingly been implicated in a diversity of chronic diseases. Accordingly, the increasing prevalence of chronic disease has underscored the importance of nutrient supplementation that may be effective in improving the metabolism and supporting overall health. Diesel particulate matter (DPM), a major contributor to urban air pollution, is composed of fine carbonaceous particles coated with organic compounds, including polycyclic aromatic hydrocarbons (PAHs) and heavy metals [1]. These particles, derived from the combustion of diesel fuel, are readily inhaled and can penetrate deep into the lungs, eliciting oxidative stress and inflammatory responses. Chronic exposure to DPM is strongly associated with respiratory diseases such as asthma, chronic obstructive pulmonary disease (COPD), and lung cancer [2]. Mechanistic studies demonstrate that DPM exposure increases levels of pro-inflammatory cytokines, such as tumor necrosis factor alpha (TNF-α) and interleukin-6 (IL-6), and oxidative stress markers including reactive oxygen species (ROS), leading to airway hyperresponsiveness and tissue remodeling [3,4]. Beyond its pulmonary effects, DPM exposure has systemic implications such as influencing metabolic health and increasing the risk of cardiovascular and endocrine diseases [5].

DPM exposure has been shown to exacerbate inflammation in adipose tissue, contributing to insulin resistance and glucose intolerance. In particular, animal studies have revealed that DPM accelerates adipocyte hypertrophy and macrophage infiltration, enhancing the production of inflammatory mediators that impair systemic insulin signaling [6]. Similarly, in skeletal muscle, DPM disrupts mitochondrial function and energy metabolism, aggravating metabolic inflexibility and oxidative damage [7]. Furthermore, hepatic responses to DPM include increased lipid accumulation and oxidative stress, accelerating the progression of non-alcoholic fatty liver disease [8].

To counteract the deleterious effects of DPM, dietary supplementation with diverse phytonutrients has gained significant attention. Among these, chlorophyllin is particularly notable for its potent antioxidant and anti-inflammatory properties. Sodium copper chlorophyllin, which is a water-soluble derivative of chlorophyll, has demonstrated a capacity to neutralize free radicals and modulate detoxification pathways, thereby reducing DNA damage and lipid peroxidation caused by environmental toxins. Unlike chlorophyll, which is fat-soluble due to its phytol tail, chlorophyllin is a semi-synthetic derivative in which the central magnesium ion is often replaced with copper or sodium, making it more hydrophilic. This structural modification allows it to dissolve easily in water, which is why chlorophyllin is commonly used in supplements, food colorants, and medicinal applications.

Studies have shown that chlorophyllin effectively attenuates oxidative damage in tissues exposed to PAHs and other carcinogenic pollutants [9,10,11]. Additionally, chlorophyllin has shown promise in enhancing hepatic detoxification processes and modulating gut microbiota to alleviate systemic inflammation [12]. In vitro and in vivo studies further highlight its ability to inhibit carcinogenesis and support cellular homeostasis [13]. Recent investigations into chlorophyll have also revealed its potential in improving mitochondrial health and reducing oxidative stress markers in metabolic tissues. Chlorophyll supplementation has been associated with improved glucose tolerance and reduced markers of systemic inflammation in preclinical studies, suggesting its efficacy in combating metabolic dysfunction [14].

This study investigated the metabolic and tissue-specific effects of DPM exposure observed in C57BL/6 mice, with specific inspection of murine lungs, adipose tissue, and skeletal muscle while evaluating the protective potential of SCC. By elucidating the molecular contributions that underpin these interactions, this research aimed to highlight practical strategies for addressing the health risks posed by air pollution.

## 2. Materials and Methods

### 2.1. Animals

Male and female C57BL/6 mice, aged 12 weeks, were housed at a temperature of 22 ± 1 °C and humidity of 60–70%. They were maintained on a 12 h light–dark cycle with free access to food. The mice were randomly divided into two groups: one with access to water (Control: CON) and the other to a supplement containing sodium copper chlorophyllin (designated as SCC hereafter; sodium copper chlorophyllin, Unicity International, Provo, UT, USA) for four weeks. Both groups had unrestrained access to standard rodent chow (LabDiet 5001, LabDiet, St. Louis, MO, USA) and their respective liquid beverage. Because 25 g mice consumed approximately 4 mL of water per day, sodium copper chlorophyllin (SCC) was mixed with water at a concentration of 3.4 µg/mL. Ensuring continuous access to this mixture as their sole hydration source led to the consumption of approximately 0.014 mg of SCC per day per 25 g mouse. This concentration and daily exposure program in the current mouse model equates to approximately 3 mg of SCC consumed daily by an average 60 kg human. Daily consumption of water and water/SCC was recorded. The mice were further divided randomly into groups exposed to room air (RA) or diesel particulate matter (DPM) for four weeks (details provided below), resulting in four groups (*n* = 8 each): CON+RA, CON+DPM, SCC+RA, and SCC+DPM. At the end of the exposure period, mice were sacrificed by researchers blinded to the mouse group or identity, and lung, gastrocnemius muscle, and adipose tissues were collected. Lung and muscle tissues were frozen and stored at −80 °C for ATP assessment or kept on ice for GSH/GSSG evaluation or mitochondrial respirometry. Adipose samples were processed for histology as previously described [15]. Blood samples were also collected for serum analysis. All studies followed the National Institutes of Health Guide for the Care and Use of Laboratory Animals and were approved by the Institutional Animal Care and Use Committee at Brigham Young University (protocol #20-0203).

### 2.2. Diesel Particulate Matter Exposure

The DPM used in these experiments was obtained from the National Institute of Standards and Technology (NIST) as Standard Reference Material (SRM) 2975. SRM-2975, originally sourced from M.E. Wright of the Donaldson Company, Inc., Minneapolis, MN, USA, was collected from a filtration system designed for diesel-powered forklifts [16]. Following collection, the DPM was homogenized and prepared as SRM-2975. Mice were placed in soft restraints and connected to the exposure tower of a nose-only exposure system (InExpose System; Scireq, Montreal, QC, Canada). Each mouse in the DPM-exposed group received a nebulized dose of 15 ng of freshly vortexed DPM in approximately 20 μL PBS, equating to roughly 3 μg/mL in plasma, a physiological dosage [17]. These controlled, precisely delivered DPM exposures lasted 30 min per day, five days a week for four weeks, totaling 20 exposures. Control animals were similarly restrained but exposed to room air (RA).

### 2.3. Tissue Permeabilization

Approximately 10–20 mg of muscle or lung tissue was kept on ice for mitochondrial respirometry. Samples were transferred to tubes containing 50 µg/mL of saponin in MiR05 respiration buffer that included 0.5 mM EGTA, 3 mM MgCl_2_, 60 mM K-lactobionate, 20 mM taurine, 10 mM KH_2_PO_4_, 20 mM HEPES, 110 mM sucrose, and 1 g/L BSA (Sigma; A3803, St. Louis, MO, USA), adjusted to pH 7.1.

### 2.4. Mitochondrial Respirometry

High-resolution oxygen consumption was measured at 37 °C in permeabilized muscle fiber bundles, adipose tissue, and liver using the Oroboros O_2_K Oxygraph with MiR05 respiration buffer (Oroboros Instruments, Innsbruck, Austria). Baseline respiration rates were determined before adding the sample to the respiration chambers. The chambers were briefly hyperoxygenated to ~250 nmol/mL, after which respiration was measured using a substrate–uncoupler–inhibitor–titration protocol. Electron flow through Complex I was supported using glutamate (10 mM) and malate (2 mM) to determine leak oxygen consumption (GM). After stabilization, ADP (2.5 mM) was added to measure oxidative phosphorylation capacity (D), followed by the addition of succinate (S) to assess Complex I + II electron flow into the Q-junction.

### 2.5. ATP Quantification

ATP levels were measured in frozen muscle or lung tissue samples using an ATPLite Luminescence Assay kit (Perkin Elmer). Frozen samples were thawed on ice and homogenized in mammalian cell lysis buffer provided by the manufacturer. Homogenates were diluted with water and transferred to opaque 96-well plates (100 µL per well). ATPLite lysis buffer (50 µL) was added to each well, and the plates were agitated at 700 rpm for 5 min at room temperature. ATPLite substrate solution (50 µL) was then added, followed by agitation for another 5 min at 700 rpm. After a 10 min dark adaptation, luminescence was measured using a Victor Nivo Multimode Plate Reader (Perkin Elmer, Waltham, MA, USA).

### 2.6. Glutathione/Glutathione Disulfide Redox Potential Analysis

The analysis of redox potential was conducted following the methods described previously [18]. Isolated tissues were collected in 5% perchloric acid and boric acid (0.2 M) containing γ-glutamylglutamate (10 μM, Sigma-Aldrich, St. Louis, MO, USA) for GSH analysis. GSH and GSSG concentrations were measured as *S*-carboxymethyl and *N*-dansyl derivatives using reverse-phase high-performance liquid chromatography. An internal standard, γ-glutamylglutamate, was used for normalization. Proteins were precipitated with acid, and samples were centrifuged at 16,000× *g* for 5 min. The soluble fraction containing free GSH and GSSG was derivatized and analyzed using an e2695 Separations Module (Waters, Milford, MA, USA) with a Supelcosil LC-NH2 5 μm column (Sigma-Aldrich). Peak detection was performed using a 2474 FLR Detector (excitation 335 nm and emission 518 nm, Waters). The GSH/GSSG redox potential (Eh) was calculated using the Nernst equation based on intracellular GSH and GSSG concentrations.

### 2.7. Serum Inflammation Assessments

Total protein in serum samples was measured using a BCA Protein Assay Kit (Thermo Fisher Scientific, Waltham, MA, USA). For each group (*n* = 8), 25 µg of protein from two animals was pooled to create a sample with a final concentration of 50 µg/mL. This process was repeated four times per group, resulting in four pooled samples per group. These samples were added to membranes from a mouse inflammation antibody array (Cat # ab13399; Abcam, Waltham, MA, USA), incubated overnight, and then retrieved for a second incubation with another antibody array membrane. Biotinylated antibodies were added to each membrane and incubated overnight, followed by incubation with a streptavidin-conjugated fluorescent label to detect cytokine expression. Membranes were imaged using a fluorescence imaging system (LI-COR) and quantified with ImageJ 1.54 (U.S. National Institutes of Health, Bethesda, MD, USA). Signal intensities were compared to positive controls on each membrane.

### 2.8. Adipose Tissue Histology and Characterization

Adipose tissue samples were collected from the inguinal and perirenal region of at least six animals per group in order to evaluate subcutaneous and visceral fat, respectively. These tissues were preserved in 4% paraformaldehyde, followed by processing, paraffin embedding, and sectioning into 5 µm slices [15]. General adipose morphology was examined using hematoxylin and eosin (H and E) staining. Adipocyte diameter was assessed using the mean linear intercept (MLI) technique, a widely applied method for estimating average distances between cellular features [19,20]. Morphological differences between diesel particulate matter (DPM)-exposed and control mice were quantified by calculating MLI values based on a point-counting approach, which involved measuring intercepts at adipocyte membranes. For each sample, at least six images were captured using an Olympus BX51 microscope with Olympus CellSense Standard 3.1 software (Bethlehem, PA, USA), and intercept counts from the images were averaged to determine group-specific means.

### 2.9. Statistical Analysis

Statistical analyses were conducted using GraphPad Prism software (version 10.1.0; GraphPad, Santa Clara, CA, USA). Mean values ± S.D. per animal group were assessed by one- and two-way analysis of variance (ANOVA), considering normal variances and variables. Mann–Whitney tests were used for additional analysis. Results with *p* ≤ 0.05 were considered significant.

## 3. Results

### 3.1. Lung Energy and Oxidative Metrics

**Figure 1:**
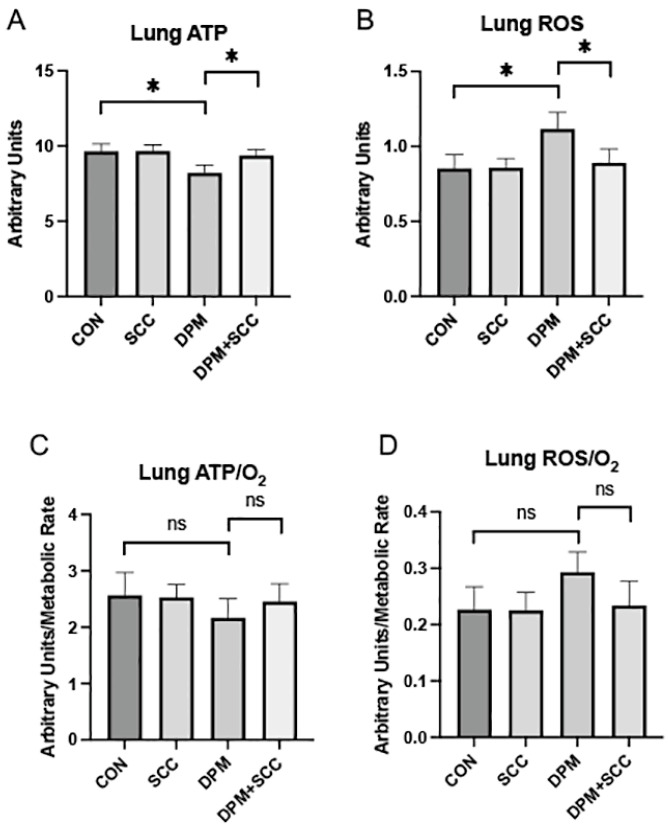
(**A**) Lung ATP levels across treatment groups. DPM treatment significantly decreased ATP synthesis compared to CON, and DPM+SCC significantly mitigated DPM-mediated ATP deficits (* *p* ≤ 0.05). (**B**) Reactive oxygen species (ROS) levels in the lung. ROS were elevated in DPM animals, and ROS in DPM+SCC returned to levels observed in CON (* *p* ≤ 0.05). (**C**) ATP/O_2_ metabolic efficiency was not significantly different (ns) when comparing any of the groups. (**D**) ROS/O_2_ levels were also not significantly different (ns) among any of the groups.

Exposure to DPM resulted in a significant decline in lung ATP levels compared to control (CON; Figure 1A, *p* ≤ 0.05). Treatment with SCC mitigated this effect, restoring ATP levels to values comparable to CON. We discovered that there was no difference in the generation of ATP in the lung when CON tissues were compared to lungs from animals with access to SCC only (Figure 1A). Levels of reactive oxygen species (ROS) were significantly elevated in DPM-exposed lungs (Figure 1B, *p* ≤ 0.05) and SCC supplementation reduced ROS levels significantly. When normalized to oxygen consumption, DPM exposure did not significantly reduce ATP/O_2_ efficiency (Figure 1C) or ROS/O_2_ output (Figure 1D).

### 3.2. Skeletal Muscle Bioenergetics and Oxidative Stress

**Figure 2:**
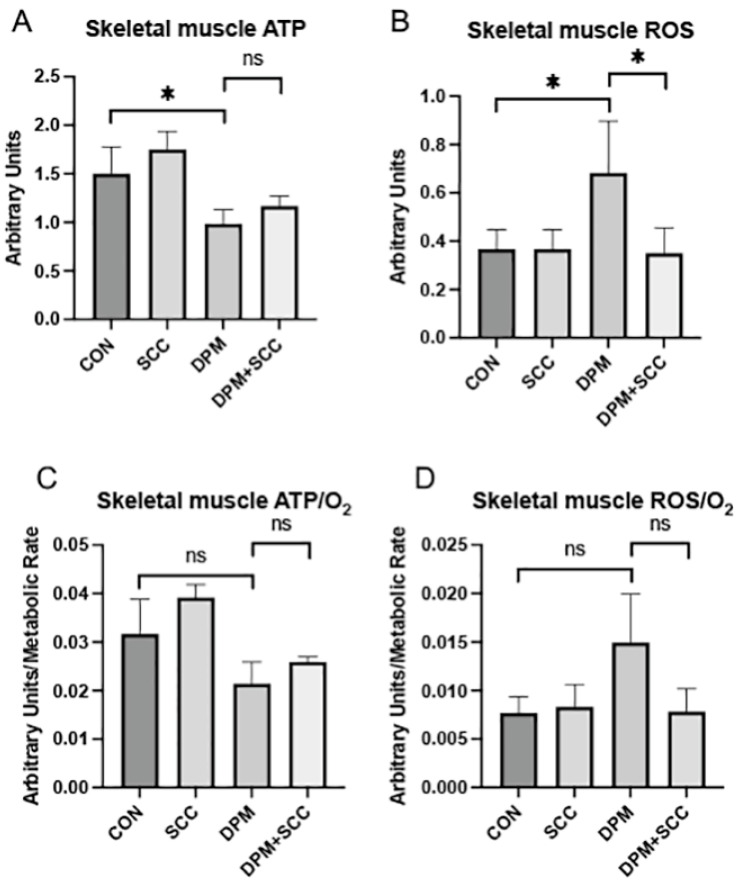
(**A**) ATP levels were significantly reduced in skeletal muscle from the DPM group compared to CON (* *p* ≤ 0.05). However, no significant difference in ATP abundance was observed when comparing DPM+SCC and DPM groups. (**B**) Skeletal muscle ROS levels were significantly elevated in DPM mice compared to CON, and DPM+SCC experienced significantly less ROS compared to the DPM group (* *p* ≤ 0.05). (**C**) Skeletal muscle ATP/O_2_ efficiency was not significant different (ns) when comparing CON vs. DPM or DPM vs. DPM+SCC. (**D**) Skeletal muscle ROS/O_2_ levels were also not significantly different (ns) when comparing CON vs. DPM or DPM vs. DPM+SCC.

ATP synthesis was markedly decreased in skeletal muscle obtained from mice exposed to DPM compared to CON (Figure 2A, *p* ≤ 0.05); however, skeletal muscle ATP synthesis for DPM-exposed mice with access to SCC was not significantly different compared to mice exposed to DPM alone (Figure 2A). ROS levels were significantly increased in DPM-exposed samples (Figure 2B, *p* ≤ 0.05), and SCC markedly reduced this ROS enhancement. Compared to CON, skeletal muscle ATP/O_2_ efficiency in DPM-exposed mice was not significantly different and SCC did not alter ATP/O_2_ ratios when compared to DPM alone (Figure 2C). ROS/O_2_ levels in DPM-exposed skeletal muscle did not increase significantly when compared to CON (Figure 2D). While DPM+SCC did not significantly lessen ROS/O_2_ when compared to DPM alone, a noteworthy trend toward mitigation was observed (Figure 2D).

### 3.3. Systemic Inflammatory Cytokines

**Figure 3:**
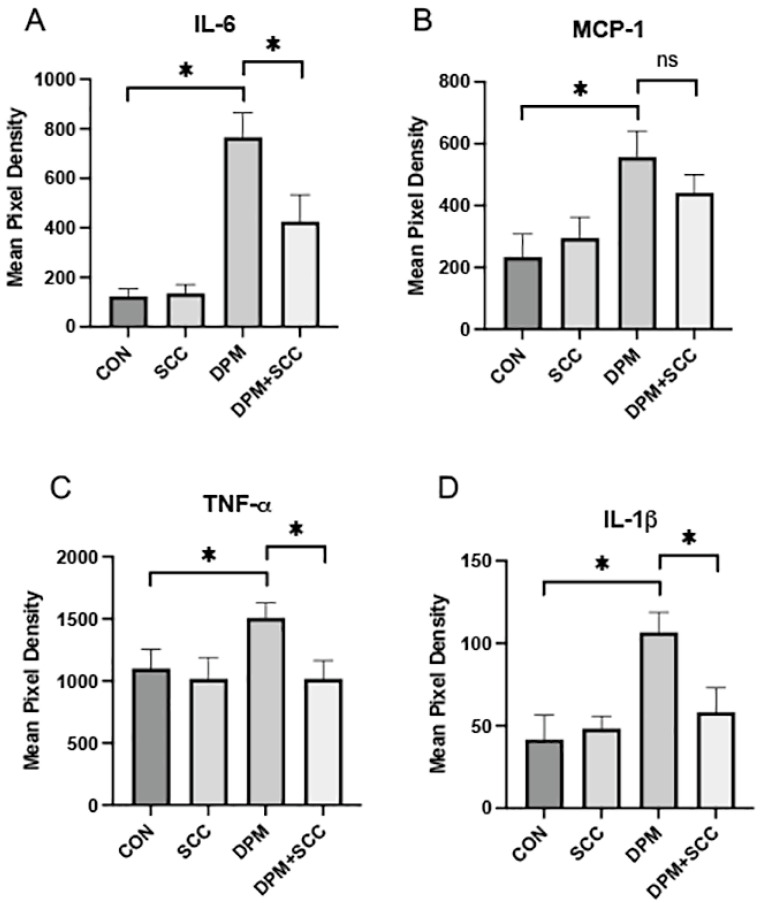
(**A**) IL-6 levels were markedly increased in DPM mice compared to CON, and DPM+SCC mice secreted significantly less IL-6 levels compared to mice exposed to DPM alone (* *p* ≤ 0.05). (**B**) MCP-1 levels were significantly elevated in DPM mice compared to CON (* *p* ≤ 0.05), and although MCP-1 levels in DPM+SCC mice trended downward, the change was not significantly different (ns). (**C**) TNF-α abundance was significantly increased in DPM mice compared to CON, and DPM+SCC significantly diminished TNF-α compared to DPM alone (* *p* ≤ 0.05). (**D**) IL-1β was significantly elevated in the DPM mice compared to CON mice, and DPM+SCC mice secreted significantly less IL-1β compared to DPM alone (* *p* ≤ 0.05).

DPM exposure significantly elevated the elaboration of inflammatory cytokines into serum, including IL-6 (Figure 3A, *p* ≤ 0.05), MCP-1 (Figure 3B, *p* ≤ 0.05), TNF-α (Figure 3C, *p* ≤ 0.05), and IL-1β (Figure 3D, *p* ≤ 0.05) when compared to room-air-exposed CON. Concomitant SCC exposure with DPM substantially reduced the expression of IL-6, TNF-α, and IL-1β (Figure 3A,C,D; each *p* ≤ 0.05) compared to the DPM alone group, indicating potential anti-inflammatory properties of SCC. MCP-1 levels in serum trended downward in DPM+SCC mice but were not significantly decreased when compared to serum obtained from DPM-exposed mice (Figure 3B).

### 3.4. Adipose Tissue Morphometry

**Figure 4:**
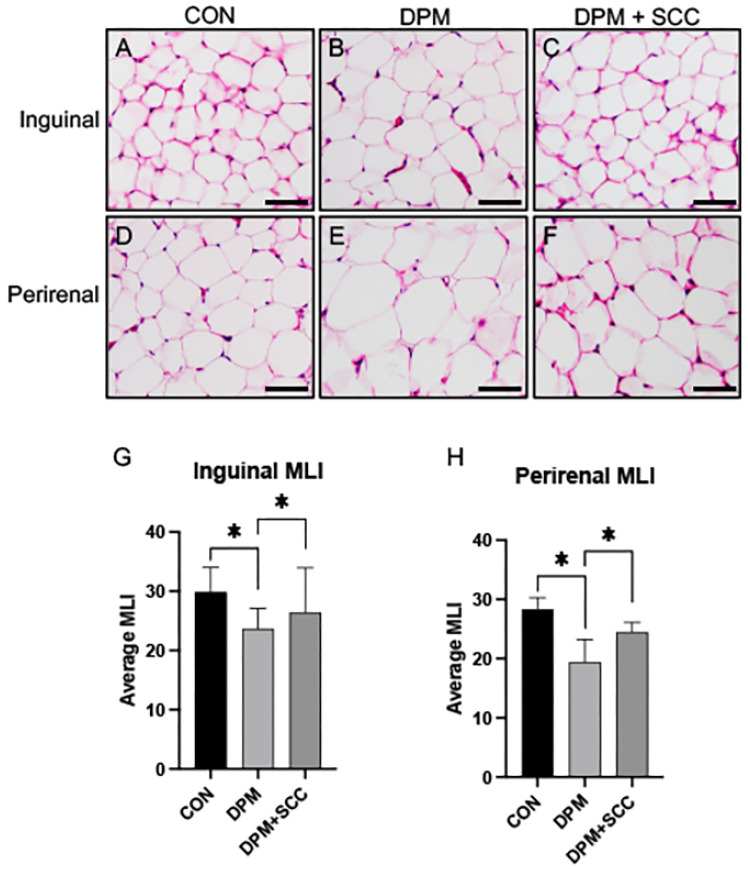
(**A**–**F**) Representative images of adipose tissue histology. Panels (**A**–**C**) display inguinal adipose tissue sections from CON, DPM, and DPM+SCC groups, respectively. Panels (**D**–**F**) display perirenal adipose tissue sections for the same groups. Morphological assessments indicate hypertrophy in DPM-treated tissues, with partial resolution in samples procured from DPM+SCC mice. (**G**) Average mean linear intercept (MLI) in inguinal adipose tissue showed significantly less intercepts, and therefore significantly larger adipocyte diameter, in DPM mice compared to CON (* *p* ≤ 0.05), and a significant attenuation in DPM+SCC samples. (**H**) Average MLI in perirenal adipose tissue revealed hypertrophic adipocytes in DPM mice compared to CON, and a significant attenuation of cellular enlargement in DPM+SCC samples (* *p* ≤ 0.05). Images are representative of 6 randomized fields obtained from *n* = 6 for each group of mice. Scale bars represent 50 mm.

Adipose tissue from the inguinal or perirenal regions of the CON animals was obtained and morphologically imaged (Figure 4A,D). Qualitative expansion of adipocytes was observed in animals exposed to DPM (Figure 4B,E). Images of fat deposits obtained from DPM-exposed animals with access to SCC show that they were detectably smaller than those in DPM-alone groups (Figure 4C,F). The average mean linear intercept (MLI) of adipocyte size in both inguinal and perirenal fat depots was significantly decreased in DPM-exposed mice compared to CON (Figure 4G,H, *p* ≤ 0.05), demonstrating adipose hypertrophy following DPM exposure. SCC supplementation significantly reduced adipocyte hypertrophy in both regions, restoring MLI to levels comparable to CON (Figure 4G,H, *p* ≤ 0.05). These data indicate that SCC may attenuate DPM-induced adipose tissue remodeling and hypertrophy.

## 4. Discussion

This study provides robust evidence for the adverse effects of diesel particulate matter (DPM) exposure on metabolic, oxidative, and inflammatory status across key tissues, including lung, skeletal muscle, and adipose depots. Further, it demonstrates the therapeutic potential of chlorophyllin (SCC) supplementation in mitigating these impacts. The findings expand on existing literature by elucidating how DPM-induced oxidative stress and inflammation disrupt systemic homeostasis and to what extent SCC ameliorates these disruptions.

Our findings align with prior studies indicating that particulate matter (PM_2.5_) exposure exacerbates adipose tissue dysfunction through increased macrophage infiltration and inflammatory cytokine production in visceral fat, a process that drives insulin resistance and metabolic dysfunction [21]. Adipocyte hypertrophy observed in both visceral (perirenal) and subcutaneous (inguinal) depots is consistent with reports of PM_2.5_-induced enlargement of adipocytes in animal models fed either standard or high-fat diets, underscoring the systemic metabolic consequences of air pollution [22]. Notably, previously published reports revealed that DPM exposure disrupts adipocyte morphology and increases oxidative stress markers in both fat types, echoing previous studies linking PM exposure to mitochondrial dysfunction and lipid metabolism alterations in adipose tissue [23]. These observations are supported by a published prospective study that investigated personal and ambient air pollution exposure and its effects on type II diabetes [24] and a more recent population-based study that examined the effects of air pollution on metabolic syndrome (MetS) prevalence and incidence [25]. Such connections between ambient air pollution and the progression of metabolic deficits are supported in epidemiological studies that involved diabetics, people with hypertension, the elderly, and others [26,27].

The significant reduction in mean linear intercept (MLI) of adipocyte size in DPM-exposed animals supplemented with SCC suggests a restorative effect on adipose architecture. This discovery aligns with evidence that chlorophyll supplementation reduces low-grade inflammation, improves glucose tolerance, and attenuates adipose tissue dysfunction in obesity models by modulating gut microbiota and pathways that govern lipid metabolism [9]. Given the critical role of visceral fat in systemic inflammation and metabolic disease, these results highlight SCC as a potential therapeutic intervention to combat air-pollution-induced adipose dysfunction.

In the lung, DPM exposure significantly increased ROS levels, reduced ATP/O_2_ efficiency, and led to elevated systemic secretion of elevated pro-inflammatory cytokines (e.g., IL-6, TNF-α, IL-1β), reinforcing prior observations of PM-induced pulmonary inflammation and oxidative damage [28]. These effects likely stem from particulate deposition in alveolar regions, where they activate inflammatory pathways and disrupt mitochondrial respiration. The ability of SCC to restore lung ATP levels and reduce ROS/O_2_ ratios underscores its efficacy in mitigating oxidative stress, potentially by neutralizing free radicals and enhancing mitochondrial efficiency. These findings are consistent with the antioxidant properties of SCC, which have been shown to reduce lipid peroxidation and DNA damage in response to environmental toxins [10].

Although skeletal muscle ATP/O_2_ efficiency was not significantly altered, DPM exposure increased ROS levels, highlighting oxidative stress as a key mediator of tissue dysfunction. SCC supplementation partially alleviated ROS levels, suggesting its role in reducing oxidative damage and preserving mitochondrial function. Reasons for these discoveries may stem from reduced substrate availability for ATP production or Complex I or Complex III damage. As such, the question to be answered relates to whether election transport slows proportionally to O_2_ consumption, causing the ATP/O_2_ ratio to remain unchanged. These findings align with studies demonstrating that PM exposure disrupts skeletal muscle energy metabolism and that antioxidant supplementation can attenuate these effects [7].

The anti-inflammatory and antioxidant properties of SCC observed across tissues indicate systemic benefits of supplementation. By reducing inflammatory cytokines such as IL-6 and TNF-α, SCC addresses a key driver of metabolic and cardiovascular diseases associated with air pollution exposure [9]. Additionally, the ability of SCC to modulate gut microbiota and improve lipid metabolism may contribute to its protective effects on adipose tissue, further reducing the risk of obesity and insulin resistance [9]. Given the global burden of air pollution, the ability of SCC to mitigate oxidative stress and inflammation across multiple tissues has profound implications for public health. Chronic exposure to PM_2.5_ is linked to increased risks of cardiovascular disease, diabetes, and respiratory conditions, making interventions that target systemic oxidative and inflammatory pathways highly desirable. SCC supplementation offers a non-invasive, dietary strategy to counteract these effects, with potential applications for at-risk populations exposed to high levels of air pollution. In summary, this study demonstrates the multifaceted impact of DPM exposure on systemic health and highlights SCC as a promising intervention to mitigate oxidative and inflammatory damage. Dissecting mechanistic control of these outcomes should naturally focus on roles mitochondria may have in reducing ROS production such as via Complex I and/or Complex III electron leakage, enhancing superoxide dismutase 2 (SOD 2) or regulating the mitochondrial permeability transition pore (mPTP) [29]. From an inflammatory standpoint, plausible roles for chlorophyllin in inhibiting NF-κB (p65/p50), particularly in light of TNF-α and IL-1β abrogation, or dissecting Nrf2/NF-κB crosstalk known to create anti-inflammatory environments would have great value [30,31].

The significance of this study opens opportunities to further explore the role such supplements may have in attenuating metabolic complications caused by particulates. However, limitations should be carefully considered in subsequent endeavors aimed at dissecting mechanisms and human relevance. Such limitations include expansion of an exposure time course, consideration of potential sex-specific responses, and absorption dynamics of SCC. Future research should accordingly explore the precise molecular mechanisms underlying these effects and assess the translational potential of SCC in human populations exposed to environmental pollutants.

## 5. Conclusions

In conclusion, this study demonstrates that diesel particulate matter (DPM) exposure induces significant metabolic and inflammatory disruptions in key tissues, including the lungs, skeletal muscle, and adipose depots, while SCC supplementation effectively mitigates these effects. SCC reduced oxidative stress, restored ATP levels, and attenuated pro-inflammatory cytokine expression, highlighting its potential as an antioxidant and anti-inflammatory intervention. These findings underscore the need for further research into SCC’s mechanistic role in mitigating pollution-induced metabolic dysfunction and its translational potential in human populations exposed to environmental pollutants.

## Data Availability

The original contributions presented in this study are included in the article. Further inquiries can be directed to the corresponding author.

## References

[1] Lazzarin M.C., Dos Santos J.F., Quintana H.T., Pidone F.A.M., de Oliveira F. (2023). Duchenne muscular dystrophy progression induced by downhill running is accompanied by increased endomysial fibrosis and oxidative damage DNA in muscle of mdx mice. J. Mol. Histol..

[2] Savary C.C., Bellamri N., Morzadec C., Langouët S., Lecureur V., Vernhet L. (2018). Long term exposure to environmental concentrations of diesel exhaust particles does not impact the phenotype of human bronchial epithelial cells. Toxicol. In Vitro.

[3] Salvi S., Blomberg A., Rudell B., Kelly F., Sandström T., Holgate S.T., Frew A. (1999). Acute inflammatory responses in the airways and peripheral blood after short-term exposure to diesel exhaust in healthy human volunteers. Am. J. Respir. Crit. Care Med..

[4] Kim D.I., Song M.K., Kim H.I., Han K.M., Lee K. (2020). Diesel Exhaust Particulates Induce Neutrophilic Lung Inflammation by Modulating Endoplasmic Reticulum Stress-Mediated CXCL1/KC Expression in Alveolar Macrophages. Molecules.

[5] Brinchmann B.C., Holme J.A., Frerker N., Rambøl M.H., Karlsen T., Brinchmann J.E., Kubátová A., Kukowski K., Skuland T., Øvrevik J. (2023). Effects of organic chemicals from diesel exhaust particles on adipocytes differentiated from human mesenchymal stem cells. Basic. Clin. Pharmacol. Toxicol..

[6] Hasegawa Y., Okamura T., Nakajima H., Kitagawa N., Majima S., Okada H., Senmaru T., Ushigome E., Nakanishi N., Hamaguchi M. (2023). Metabolic outcomes and changes in innate immunity induced by diesel exhaust particles airway exposure and high-fat high-sucrose diet. Life Sci..

[7] Tomaru M., Takano H., Inoue K., Yanagisawa R., Osakabe N., Yasuda A., Shimada A., Kato Y., Uematsu H. (2007). Pulmonary exposure to diesel exhaust particles enhances fatty change of the liver in obese diabetic mice. Int. J. Mol. Med..

[8] Ding S., Yuan C., Si B., Wang M., Da S., Bai L., Wu W. (2019). Combined effects of ambient particulate matter exposure and a high-fat diet on oxidative stress and steatohepatitis in mice. PLoS ONE.

[9] Li Y., Cui Y., Hu X., Liao X., Zhang Y. (2019). Chlorophyll Supplementation in Early Life Prevents Diet-Induced Obesity and Modulates Gut Microbiota in Mice. Mol. Nutr. Food Res..

[10] Møller P., Daneshvar B., Loft S., Wallin H., Poulsen H.E., Autrup H., Ravn-Haren G., Dragsted L.O. (2003). Oxidative DNA damage in vitamin C-supplemented guinea pigs after intratracheal instillation of diesel exhaust particles. Toxicol. Appl. Pharmacol..

[11] Flury M., Mathison J.B., Harsh J.B. (2002). In situ mobilization of colloids and transport of cesium in Hanford sediments. Environ. Sci. Technol..

[12] Zheng H., You Y., Hua M., Wu P., Liu Y., Chen Z., Zhang L., Wei H., Li Y., Luo M. (2018). Chlorophyllin Modulates Gut Microbiota and Inhibits Intestinal Inflammation to Ameliorate Hepatic Fibrosis in Mice. Front. Physiol..

[13] Weickert M.O., Pfeiffer A.F. (2008). Metabolic effects of dietary fiber consumption and prevention of diabetes. J. Nutr..

[14] Yang Y., Jiang X., Pandol S.J., Han Y.P., Zheng X. (2021). Green Plant Pigment, Chlorophyllin, Ameliorates Non-alcoholic Fatty Liver Diseases (NAFLDs) Through Modulating Gut Microbiome in Mice. Front. Physiol..

[15] Warren C.E., Campbell K.M., Kirkham M.N., Saito E.R., Remund N.P., Cayabyab K.B., Kim I.J., Heimuli M.S., Reynolds P.R., Arroyo J.A. (2024). The Effect of Diesel Exhaust Particles on Adipose Tissue Mitochondrial Function and Inflammatory Status. Int. J. Mol. Sci..

[16] Ahmed T.M., Bergvall C., Åberg M., Westerholm R. (2015). Determination of oxygenated and native polycyclic aromatic hydrocarbons in urban dust and diesel particulate matter standard reference materials using pressurized liquid extraction and LC-GC/MS. Anal. Bioanal. Chem..

[17] Barton D.B., Betteridge B.C., Earley T.D., Curtis C.S., Robinson A.B., Reynolds P.R. (2014). Primary alveolar macrophages exposed to diesel particulate matter increase RAGE expression and activate RAGE signaling. Cell Tissue Res..

[18] Walton C.M., Saito E.R., Warren C.E., Larsen J.G., Remund N.P., Reynolds P.R., Hansen J.M., Bikman B.T. (2023). Yerba Maté (Ilex paraguariensis) Supplement Exerts Beneficial, Tissue-Specific Effects on Mitochondrial Efficiency and Redox Status in Healthy Adult Mice. Nutrients.

[19] Hsia C.C., Hyde D.M., Ochs M., Weibel E.R. (2010). An official research policy statement of the American Thoracic Society/European Respiratory Society: Standards for quantitative assessment of lung structure. Am. J. Respir. Crit. Care Med..

[20] Martinez-Santibanez G., Singer K., Cho K.W., DelProposto J.L., Mergian T., Lumeng C.N. (2015). Obesity-induced remodeling of the adipose tissue elastin network is independent of the metalloelastase MMP-12. Adipocyte.

[21] Mendez R., Zheng Z., Fan Z., Rajagopalan S., Sun Q., Zhang K. (2013). Exposure to fine airborne particulate matter induces macrophage infiltration, unfolded protein response, and lipid deposition in white adipose tissue. Am. J. Transl. Res..

[22] Xu X., Yavar Z., Verdin M., Ying Z., Mihai G., Kampfrath T., Wang A., Zhong M., Lippmann M., Chen L.C. (2010). Effect of early particulate air pollution exposure on obesity in mice: Role of p47phox. Arterioscler. Thromb. Vasc. Biol..

[23] Xu X., Liu C., Xu Z., Tzan K., Zhong M., Wang A., Lippmann M., Chen L.C., Rajagopalan S., Sun Q. (2011). Long-term exposure to ambient fine particulate pollution induces insulin resistance and mitochondrial alteration in adipose tissue. Toxicol. Sci..

[24] Sun Z., Mukherjee B., Brook R.D., Gatts G.A., Yang F., Sun Q., Brook J.R., Fan Z., Rajagopalan S. (2013). Air-Pollution and Cardiometabolic Diseases (AIRCMD): A prospective study investigating the impact of air pollution exposure and propensity for type II diabetes. Sci. Total Environ..

[25] Matthiessen C., Lucht S., Hennig F., Ohlwein S., Jakobs H., Jöckel K.H., Moebus S., Hoffmann B. (2018). Long-term exposure to airborne particulate matter and NO(2) and prevalent and incident metabolic syndrome—Results from the Heinz Nixdorf Recall Study. Environ. Int..

[26] Hwang M.J., Kim J.H., Koo Y.S., Yun H.Y., Cheong H.K. (2020). Impacts of ambient air pollution on glucose metabolism in Korean adults: A Korea National Health and Nutrition Examination Survey study. Environ. Health.

[27] Zhang Y., Xia Y., Chang Q., Ji C., Zhao Y., Zhang H. (2023). Exposure to ambient air pollution and metabolic kidney diseases: Evidence from the Northeast China Biobank. Nephrol. Dial. Transplant..

[28] Pan K., Jiang S., Du X., Zeng X., Zhang J., Song L., Zhou J., Kan H., Sun Q., Xie Y. (2019). AMPK activation attenuates inflammatory response to reduce ambient PM(2.5)-induced metabolic disorders in healthy and diabetic mice. Ecotoxicol. Environ. Saf..

[29] Zhao R.Z., Jiang S., Zhang L., Yu Z.B. (2019). Mitochondrial electron transport chain, ROS generation and uncoupling (Review). Int. J. Mol. Med..

[30] Sandberg M., Patil J., D’Angelo B., Weber S.G., Mallard C. (2014). NRF2-regulation in brain health and disease: Implication of cerebral inflammation. Neuropharmacology.

[31] Bivol L.M., Berge R.K., Iversen B.M. (2008). Tetradecylthioacetic acid prevents the inflammatory response in two-kidney, one-clip hypertension. Am. J. Physiol. Regul. Integr. Comp. Physiol..

